# Group B Streptococcus and HIV Infection in Pregnant Women, Malawi, 2008–2010

**DOI:** 10.3201/eid1710.102008

**Published:** 2011-10

**Authors:** Katherine J. Gray, George Kafulafula, Mary Matemba, Mercy Kamdolozi, Gladys Membe, Neil French

**Affiliations:** Author affiliations: College of Medicine, Blantyre, Malawi (K.J. Gray, G. Kafulafula, M. Kamdolozi, G. Membe);; Karonga Prevention Study, Karonga, Malawi (M. Matemba, N. French);; London School of Hygiene and Tropical Medicine, London, UK (N. French)

**Keywords:** HIV, HIV and pregnancy, Africa, Group B streptococcus, bacteria, carriage, cross-sectional study, virus, dispatch

## Abstract

To determine whether an association exists between group B streptococcus carriage and HIV infection, we recruited 1,857 pregnant women (21.7% HIV positive) from Queen Elizabeth Central Hospital, Blantyre, Malawi. Overall, group B streptococcus carriage was 21.2% and did not differ by HIV status. However, carriage was increased among HIV-positive women with higher CD4 counts.

Group B streptococcus (GBS) is the major cause of neonatal meningitis and septicemia in Malawi, a problem that has only recently been recognized (*1*). A similar pattern of disease has emerged from other countries in eastern and southern Africa (*2**–**8*). The geographic distribution of these reports led us to speculate that an association exists between GBS and HIV infection. Carriage of GBS is a prerequisite for the development of early-onset neonatal GBS disease (*9*) and is a convenient endpoint for a cross-sectional study to assess the interaction of GBS and HIV.

## The Study

Pregnant women (≥16 years of age) in their third trimester of pregnancy were recruited from the labor ward of Queen Elizabeth Central Hospital, Blantyre (QECH), during October 2008–March 2010. Recruitment was performed by a single study midwife on Sunday–Thursday each week from 7:30 am to 4:00 pm, when the midwife was not required to provide emergency care. Women were excluded if they were deemed to be too sick by the study midwife. Following the patient’s signed informed consent, medical and reproductive history was recorded, low vaginal and rectal swab specimens were obtained, and after appropriate counseling, a blood sample was obtained to either confirm or determine HIV status. CD4 counts were determined for those who were HIV positive. In accordance with Ministry of Health Guidelines, prevention of mother-to-child transmission care (PMTCT) was offered to women who were HIV infected (*10*); PMTCT was adapted as appropriate to the woman’s CD4 count. The mothers were not followed after they left the hospital.

Swab specimens were placed into Todd-Hewitt broth (Oxoid Ltd, Basingstoke, UK) supplemented with 15 μg/mL nalidixic acid and 10 μg/mL colistin and then incubated for 18–24 h. GBS were identified by phenotypic characteristics, CAMP test, and serologic analysis (Oxoid). Serotyping of GBS was performed by use of a commercial serotyping kit (Statens Serum Institut, Copenhagen, Denmark). HIV testing was conducted by using a method based on rapid tests (*11*).

The study aimed to recruit 1,950 women to show a GBS carriage prevalence increased by ≥40% in the HIV-positive women at a significance level of 5% and power of 80%. This assumed 20% HIV prevalence in the labor ward attendees (thus a 4:1 ratio of HIV-negative to HIV-positive women) and 15% GBS carriage in HIV-negative women. Two analyses were planned a priori. The primary analysis was to compare GBS carriage prevalence by HIV status; the secondary analysis was the association of GBS carriage by CD4 count in HIV-infected women. The study was approved by the College of Medicine Research and Ethics Committee of the University of Malawi and the Ethics Committee of the London School of Hygiene and Tropical Medicine.

A total of 16,456 women attended QECH labor ward during the study period. Of these women, 11,861 attended on a recruitment day, and 8,099 attended during recruitment hours, of whom 1,857 (23%) were recruited into the study (Table 1). The 2 primary reasons for not enrolling women were that they were in the second stage of labor and that their hospitalization was caused by an emergency or due to a complicated pregnancy (50.8% of all attendees). The remaining nonrecruits were women who could not be assessed by the study midwife within the specified time. During the same period, 14,783 total deliveries took place at QECH, with 380 multiple births (2.6%), 2,962 caesarean sections (20.0%), 514 stillbirths (3.5%), 29 neonatal deaths, and 20 maternal deaths recorded on the labor ward. The percentage of women in the study who had multiple births (2.7%) and caesarean sections (20.6%) was the same as that in the larger group attending the labor ward. However, the percentage with stillbirths (1.6%) was less, which is consistent with the exclusion of women who were attending the ward for complicated pregnancies or emergency medical conditions.

**Table 1:** Comparison of variables, including age, CD4 counts, and reproductive and medical history, for HIV-negative and HIV-positive study participants, Malawi, 2008–2010*

Variable	All study participants, N = 1,857†	HIV-positive participants, n = 402/21.7%	HIV-negative participants, n = 1,454/78.3%	p value
Mean age, y (SD)	25.2 (5.9)	26.7 (5.5)	24.8 (5.9)	<0.001
No. (%) recruited during wet season (Nov–Mar)	1,057 (57.0)	234 (58.2)	820 (56.4)	0.57
Reproductive and medical history				
Pregnancies, median no. (range)	1 (0–10)	2 (0–8)	1 (0–10)	<0.001
Live births, median no. (range)	1 (0–10)	1 (0–7)	1 (0–10)	<0.001
Previous neonatal death, no. (%)	45 (2.4)	11 (2.7)	34 (2.3)	0.65
Median time since LMP, days (range)	275 (98–371)	275 (145–371)	276 (98–370)	0.17
Unwell at labor ward attendance, no. (%)	114 (6.1)	33 (8.2)	81 (5.6)	0.05
Membranes ruptured >18 h before enrolled, no. (%)	52 (2.8)	12 (3.0)	40 (2.8)	0.79
Previous HIV test, no. (%)	1,664 (89.7)	368 (91.5)	1,296 (89.1)	0.16
Taking ART, no. (%)	–	83 (20.7)	–	
Median duration of ART, mo (range)		4 (1–144)		
Genital ulcer disease, no. (%)	46 (2.5)	32 (8.0)	14 (1.0)	<0.001
Sexually transmitted disease, no. (%)	34 (1.8)	23 (5.7)	11 (0.8)	<0.001
S-P use during last 4 wk of pregnancy, no. (%)	1,779 (96.0)	379 (94.3)	1,395 (96.9)	0.38
Examination and other findings				
First stage of labor, no. (%)	1,298 (69.9)	279 (69.4)	1,019 (70.1)	0.79
Premature labor before 36 wk, no. (%)	270 (14.5)	71 (17.7)	199 (13.7)	0.04
Registered for PMTCT on labor ward, no. (%)	–	333 (82.8)‡	–	
Mean birth weight (SD), kg	2.90 (0.54)	2.81 (0.58)	2.91 (0.52)	<0.001
Delivery by cesarean section, no. (%)	383 (20.6)	76 (18.9)	307 (21.1)	0.33
Stillbirth, no. (%)	30 (1.6)	13 (3.2)	17 (1.2)	0.004
Multiple birth, no. (%)	50 (2.7)	10 (2.5)	40 (2.8)	0.77
Laboratory tests				
Plasma sample for CD4 count, no.	–	383§	–	
CD4 count, median (range)		370 (20–1595)		
No. failed samples¶	18	5	13	
GBS isolated, no. (%)#	390 (21.2)	77 (19.4)	313 (21.5)	0.4
Serotype				
1a	71 (18.2)	11 (14.3)	60 (19.2)	
1b	24 (6.2)	1 (1.3)	23 (7.4)	
2	40 (10.3)	9 (11.7)	31 (9.9)	
3	152 (39.0)	32 (41.6)	120 (38.3)	
4	1 (0.3)	0	1 (0.3)	
5	93 (23.9)	23 (29.9)	70 (22.4)	
6 (1 sample), 8 (2 samples)	3 (0.8)	0	3 (1.0)	
Untypeable, no (%)	6 (1.5)	1 (1.3)	5 (1.6)	

*LMP, last menstrual period; ART, antiretroviral treatment; –, not applicable; S-P, sulfadoxine-pyrimethamine; PMTCT, prevention of mother-to-child transmission care; GBS, group B streptococcus. †Form for 1 participant was missing. ‡No. includes only participants confirmed as HIV-positive. §15 failures, 4 samples not obtained. ¶Contamination of the selective broth in 9 cases and incubator failure in 9 cases. #Failed samples excluded from denominator to derive percentage.

Of the study participants with HIV, >80% had been tested before attending QECH labor ward, most as a part of the PMTCT process. Only 125 (31%) of those with positive test results had received any formal HIV clinic care. GBS carriage was detected in 21.7% of the HIV-negative women and 19.4% of the HIV-positive women (χ^2^ = 0.99, p = 0.32) (Table 2). In the HIV-positive women, a difference in GBS carriage was noted by CD4 level: women with CD4 counts >500 cells/mm^3^ were >2× more likely than those with counts <200 cells/mm^3^ to be GBS carriers. When adjusted for antiretroviral treatment, the number of children previously borne, and age, this finding persisted (Table 2). Carriage in the HIV-infected group with CD4 counts >500 cells/mm^3^ (28.2%) was higher than in the HIV-uninfected women (21.7%), but the difference did not reach significance in an unadjusted comparison (χ^2^ = 2.65, df = 1, p = 0.11).

**Table 2:** Associations with GBS carriage among 340 pregnant women with and without HIV infection, Malawi, 2008–2010*

Variable	GBS carriage, no. positive for variable/no. total (%)	OR (95% CI)	Adjusted OR (95% CI)
All study participants	390/1,857 (21.0)	–	–
HIV-negative participants	313/1,441 (21.7)	Reference	Reference
HIV-positive participants	77/397 (19.4)	0.88 (0.66–1.17)	0.84 (0.63–1.12)
Age group, y†			
16–19	68/345 (19.7)	2.29 (0.68–7.76)	1.41 (0.40–4.92)
20–24	122/582 (21.0)	2.48 (0.74–8.28)	1.96 (0.58–6.63)
25–29	113/498 (22.7)	2.74 (0.82–9.18)	2.43 (0.72–8.18)
30–34	60/261 (23.0)	2.79 (0.82–9.48)	2.59 (0.76–8.88)
35–39	24/122 (19.7)	2.29 (0.64–8.15)	2.03 (0.56–7.33)
>40	3/31 (9.7)	Reference	Reference
First pregnancy	137 (24.0)	1.30 (1.01–1.65)	1.62 (1.19–2.19)
Second or subsequent pregnancy	244 (19.6)	Reference	Reference
Recruited during rainy season	235 (22.4)	1.18 (0.94–1.50)	–
Recruited during nonrainy season	155 (19.6)	Reference	–
Birth weight <2.5 kg			
No	307 (21.5)	Reference	–
Yes	71 (19.4)	0.88 (0.65–1.17)	–
Multiple birth			
No	381 (21.3)	Reference	–
Yes	9 (18.0)	0.81 (0.34–1.71)	–
HIV-positive subgroup‡			
Antiretroviral treatment			
No	67 (20.4)	Reference	Reference
Yes	15 (18.1)	0.91 (0.45–1.75)	1.14 (0.58–2.26)§
CD4 cell count/mm^3^¶			
<200	9 (14.1)	Reference	Reference
200–499	34 (17.2)	1.27 (0.57–2.81)	1.30 (0.58–2.92)§
>500	33 (28.2)	2.40 (1.07–5.41)	2.55 (1.10–5.90)

*GBS, group B streptococcus; OR, odds ratio; CI, confidence interval; –, not applicable. Adjusted OR derived from logistic regression by use of backward stepwise approach. Final model retained variables with p value <0.1 along with age group and parity because of their known association with GBS carriage. †χ^2^ test for linear trend, p = 0.93. ‡χ^2^ test for linear trend, p = 0.01. §Adjusted model includes age group, first pregnancy vs. second or subsequent pregnancies; antiretroviral treatment, and CD4 count for the HIV-positive subgroup. ¶19 participants had no CD4 count available.

To test for unrecorded cotrimoxazole use as an explanation of the CD4-associated carriage findings (i.e., that sicker women with more advanced disease might be more likely to take cotrimoxazole), we performed a post-hoc subgroup analysis on women who had reported no previous HIV testing or care before their recent PMTCT test, considering it unlikely that they would be taking cotrimoxazole. In this group of 277 women (13 with missing CD4 counts), the trend for increasing odds of carriage with higher CD4 count persisted. The prevalence of carriage in the CD4 groups of <200, 200–500, and >500 cells/mm^3^ was 15.9%, 19.1% (odds ratio [OR] 1.25, 200–500 cells/mm^3^ vs. <200 cells/mm^3^), and 31.0% (OR 2.37, >500 cells/mm^3^ vs. <200 cells/mm^3^), respectively (p = 0.03, by χ^2^ test for linear trend). This pattern was similar in the 125 participants who reported accessing HIV care, with ORs of 1.6 and 2.8 in the same CD4 group comparisons.

## Conclusions

In the primary analysis comparing carriage prevalence by HIV status, no overall difference in GBS carriage by HIV status was detected. The overall carriage frequency of GBS of ≈20% is comparable with those in other reports from Africa and the industrialized world. However, in the subgroup analysis of HIV-positive women, contrary to our expectations, GBS carriage was significantly increased at higher CD4 counts. Unrecorded use of antimicrobial drugs, particularly cotrimoxazole prophylaxis, as a confounder for this association was considered and dismissed as an explanation for these findings.

Antiretroviral treatment was not shown as an independent risk factor for carriage, but the cross-sectional design of this study precludes any firm conclusions. With increasing numbers of HIV-positive women using antiretroviral drugs, the effect of treatment-induced improvements in CD4 count and the potential for increased GBS carriage merit further investigation.

Our results showed a trend toward higher GBS carriage in HIV-infected women with CD4 counts >500 cells/mm^3^ than in the HIV-uninfected women. This association may be consistent with a GBS-specific immune defect, which would concur with what we understand about HIV immunopathology and related capsulate bacteria (*12*). We propose that this higher carriage is obscured at lower CD4 counts by competitive exclusion of GBS in the vagina of women with advanced HIV as a consequence of ecologic changes in the microbial flora (*13*). Increased presence of bacterial vaginosis and anaerobes at low CD4 counts is a feature of HIV, and these conditions may alter the ability of GBS to colonize the vagina (*14**,**15*). Specific studies to investigate anti-GBS immunity and the interactions of the microbial flora in HIV-infected women are required.

Neonatal GBS disease is common in Africa, and disease risk is intimately connected to GBS carriage. The public health consequences of these carriage findings are unclear at present, but further investigation of the interaction of HIV and GBS carriage and risk of neonatal disease is merited, given the recent rise in frequency of GBS infection.
